# STIM1 at the plasma membrane as a new target in progressive chronic lymphocytic leukemia

**DOI:** 10.1186/s40425-019-0591-3

**Published:** 2019-04-23

**Authors:** Marjolaine Debant, Miguel Burgos, Patrice Hemon, Paul Buscaglia, Tinhinane Fali, Sarra Melayah, Nelig Le Goux, Christophe Vandier, Marie Potier-Cartereau, Jacques-Olivier Pers, Adrian Tempescul, Christian Berthou, Cristina Bagacean, Olivier Mignen, Yves Renaudineau

**Affiliations:** 1 INSERM U1227 B lymphocytes and autoimmunity, University of Brest, Brest, France; 2 INSERM U1069, N2C, 37032, University of Tours, Tours, France; 3 IC-CGO network from “Canceropole Grand Ouest”, Brest, France; 4 Laboratory of Immunology and Immunotherapy, CHRU Brest Morvan, Brest, France; 5 Department of Haematology, CHRU Brest Morvan, Brest, France

**Keywords:** CLL, STIM1, Constitutive Ca^2+^ entry, Disease outcome

## Abstract

**Background:**

Dysregulation in calcium (Ca^2+^) signaling is a hallmark of chronic lymphocytic leukemia (CLL). While the role of the B cell receptor (BCR) Ca^2+^ pathway has been associated with disease progression, the importance of the newly described constitutive Ca^2+^ entry (CE) pathway is less clear. In addition, we hypothesized that these differences reflect modifications of the CE pathway and Ca^2+^ actors such as Orai1, transient receptor potential canonical (TRPC) 1, and stromal interaction molecule 1 (STIM1), the latter being the focus of this study.

**Methods:**

An extensive analysis of the Ca^2+^ entry (CE) pathway in CLL B cells was performed including constitutive Ca^2+^ entry, basal Ca^2+^ levels, and store operated Ca^2+^ entry (SOCE) activated following B cell receptor engagement or using Thapsigargin. The molecular characterization of the calcium channels Orai1 and TRPC1 and to their partner STIM1 was performed by flow cytometry and/or Western blotting. Specific siRNAs for Orai1, TRPC1 and STIM1 plus the Orai1 channel blocker Synta66 were used. CLL B cell viability was tested in the presence of an anti-STIM1 monoclonal antibody (mAb, clone GOK) coupled or not with an anti-CD20 mAb, rituximab. The Cox regression model was used to determine the optimal threshold and to stratify patients.

**Results:**

Seeking to explore the CE pathway, we found in untreated CLL patients that an abnormal CE pathway was (i) highly associated with the disease outcome; (ii) positively correlated with basal Ca^2+^ concentrations; (iii) independent from the BCR-PLCγ2-InsP_3_R (SOCE) Ca^2+^ signaling pathway; (iv) supported by Orai1 and TRPC1 channels; (v) regulated by the pool of STIM1 located in the plasma membrane (STIM1_PM_); and (vi) blocked when using a mAb targeting STIM1_PM_. Next, we further established an association between an elevated expression of STIM1_PM_ and clinical outcome. In addition, combining an anti-STIM1 mAb with rituximab significantly reduced in vitro CLL B cell viability within the high STIM1_PM_ CLL subgroup.

**Conclusions:**

These data establish the critical role of a newly discovered BCR independent Ca^2+^ entry in CLL evolution, provide new insights into CLL pathophysiology, and support innovative therapeutic perspectives such as targeting STIM1 located at the plasma membrane.

**Electronic supplementary material:**

The online version of this article (10.1186/s40425-019-0591-3) contains supplementary material, which is available to authorized users.

## Background

Chronic lymphocytic leukemia (CLL) is characterized by a heterogeneous natural history that is partly predicted by clinical, epigenetic and genetic features [1]. In addition, there is long standing evidence that CLL B cells (B-CLL) present an altered calcium (Ca^2+^) signaling pathway, which evolves with disease progression [2, 3]. Induction of the Ca^2+^ signaling pathway in B cells is thought to follow the model in which BCR interaction with the antigen results in the formation of the signalosome consisting of an active complex composed of spleen tyrosine kinase (Syk), B-cell linker protein (BLNK), Bruton-tyrosine-kinase (BTK), phospholipase C gamma 2 (PLCγ2), and phosphatidylinositol-4,5-bisphosphate 3-kinase δ (PI3Kδ) (Additional file 1: Figure S1A). Signalosome activation cleaves the membrane phospholipid phosphatidyl inositol 4.5-biphosphate (InsP2) into diacylglycerol (DAG) and inositol 1,4,5-triphosphate (InsP3), which subsequently, through binding to the endoplasmic reticulum (ER) IP3 receptor (InsP_3_R), mobilizes initially Ca^2+^ from stores and secondarily extracellular Ca^2+^ [4]. The reticular stromal interaction molecule 1 (STIM1) and plasma-membrane Orai1 channel are believed to be the main molecular actors of store operated Ca^2+^ entry (SOCE) in lymphocytes [5–8]. Finally, an increase in Ca^2+^ entry following B cell receptor (BCR) engagement leads to cellular metabolic changes, cell survival, proliferation, differentiation, migration, antibody production and, at very high concentrations, apoptosis [9–12].

At first glance, CLL cases with indolent and stable disease present B cells that are ineffective at mobilizing Ca^2+^ after BCR cross-linking, thus resembling B cells anergized in vivo after chronic antigenic stimulation [13]. For these patients, B-CLL cell incapacity to mobilize Ca^2+^ was related to mutated *IgHV* patients, a reduced level of cell surface (s) IgM, and a defective signalosome. In contrast, CLL cases with a worse clinical outcome show an elevated basal Ca^2+^ level that can be enhanced upon sIgM triggering. The elevated Ca^2+^ signaling in the CLL group with progressive disease was associated with an unmutated *IgHV* status and an elevated level of CD38, but was not linked to any specific cytogenetic markers [14]. However, other processes are described in order to provide alternative explanations for Ca^2+^ dysregulation in B-CLL cells such as a BCR autonomous signaling capacity due to an internal epitope present in the second framework of stereotyped *IgHV* that can be abrogated by using a BCR signaling inhibitor [15], an incapacity of the ER to release Ca^2+^ due to an inhibitory interaction between Bcl-2 (overexpressed in B-CLL cells) and the endoplasmic InsP_3_R [16], and last but not least an incompletely characterized BCR independent Ca^2+^ pathway recently described in B-CLL cells [17, 18]. Ca^2+^ deregulations in B-CLL cells and their correlation with disease evolution and severity are far from being fully understood. Reversing specific changes in deregulated Ca^2+^ fluxes may also represent new therapeutic opportunities to answer unmet needs in CLL treatment.

In this study we deciphered Ca^2+^ entry deregulation in B-CLL cells and tested whether BCR-dependent or BCR-independent Ca^2+^ entry would be relevant in CLL outcome. The latter was critical for disease progression, and we therefore analyzed and characterized a novel Ca^2+^ signaling pathway, referred to as constitutive Ca^2+^ entry (CE), which is triggered by STIM1 located at the plasma-membrane (STIM1_PM_). Interestingly, we demonstrated that blocking CE with an anti-STIM1 monoclonal antibody (mAb) presents innovative therapeutic perspectives in CLL.

## Materials and methods

### CLL population

Clinical information was retrospectively obtained from 74 untreated patients diagnosed with CLL according to the World Health Organization (WHO) classification [19], and 13 healthy volunteers at the Brest University Hospital. Disease assessment included Binet stage determination, progression free survival (PFS), treatment free survival (TFS), CD38 expression, lymphocyte counts, lymphocyte doubling time (LDT), cytogenetic risk-status, and *IgHV* mutational status, which were performed as previously described [20]. Consent was obtained from all individuals and the protocol approved by the Ethical Board at the Brest University Hospital (clinicaltrials: NCT03294980; cohort OFICE; CRB Biobank collection 2008–2014), in accordance with the Declaration of Helsinki.

### Sample preparation and flow cytometry

Peripheral blood mononuclear cells (PBMC) were isolated from whole blood by Ficoll-Hypaque density gradient centrifugation (Eurobio, Courtaboeuf, France) and B cells were further enriched using the Pan B-cell Isolation Kit (Miltenyi Biotec GmbH, Bergisch Gladbach, Germany). Cell purity was assessed by fluorescence-activated cell sorting (FACS) analysis and was over 95% for B cells (CD19+).

All monoclonal antibodies (mAb) were from Beckman-Coulter (Brea, CA, USA) unless specified: FITC-conjugated anti-IgM (clone: SA-D4), cyanin (Cy)5.5-conjugated anti-CD38 (LS198.4.3), electron coupled dye (ECD)-labelled anti-CD5 (BL1a), allophycocyanin (APC)-conjugated anti-IgD (1A6–2, BD Biosciences, Franklin-Lakes, NJ), APC-Alexa Fluor 700 (AF700)-conjugated anti-CD19 (J4–119), Pacific blue (PB)-conjugated anti-CD21 (1A4CD27) mAbs with saturating concentrations were incubated for 10 min at RT with 10 μl of PBS-washed blood. Versalyse solution (Beckman- Coulter) was added for 10 min in order to lyse red blood cells. The determination of the mean fluorescence intensity (MFI) of all markers required a minimum of 5000 events. The results were standardized to those obtained with isotype controls. Data were analysed using Kaluza 1.5 software (Beckman-Coulter).

For intracellular staining, preliminary fixation and permeabilization were performed with the cytofix/cytoperm kit (BD Biosciences) followed by a 30 min incubation at 4 °C with Phyco-Erythrin (PE)-conjugated anti-STIM1 (Gok, BD Biosciences), PC7-conjugated anti-CD19 and APC-AF700-conjugated anti-CD5 mAbs. The same protocol was applied for plasma membrane STIM1_PM_ with the omission of the permeabilization step. In the transfection experiments a polyclonal rabbit anti-human Orai1 Ab (Sigma-Aldrich, Saint-Louis, MO) or anti-TRPC1 Ab (Alomone, Jerusalem, Israel) were used after the permeabilization step, and mAb staining was assessed with a FITC-conjugated goat anti-rabbit IgG (Jackson Immunoresearch, Ely, UK). To assess PLCγ2 phosphorylation, 5 × 10^5^ cells were fixed and permeabilized with cold 80% methanol solution during 30 min. After 2 washes in PBS-BSA 0.05%, APC-conjugated anti-pPLCγ2 mAb (clone K86–689.37, BD Biosciences) was added.

### Effect on cell viability

For each patient, B-CLL cells were incubated 48 h in 24-well plates at 37 °C with either (i) 10 μg/ml murine IgG2a (Biolegend, San-Diego, CA) (ii) 10 μg/ml anti-STIM1 (Gok), (iii) 10 μg/ml RTX (Roche, Paris, France), or (iv) 2x10μg/ml anti-STIM1 + RTX. All cultures were set at 5 × 10^5^ B cells/mL in RPMI-1640 (Sigma-Aldrich) supplemented with 2 mM L-glutamine, antibiotics, and 20% of decomplemented normal human serum AB (Invitrogen, Carlsbad, CA). B cells were recovered, washed and stained for 15 min with FITC-conjugated annexin-V (AV) / propidium iodide (PI) and AF700-conjugated anti-CD19 antibody according to the Beckman-Coulter apoptosis kit protocol. The decrease in the percentage of live cells (CD19^+^ AV-PI-) was recorded.

### Calcium entry recording

For CE measurements, B cells were loaded with 2 μM Fura-2/AM dye (Molecular Probes, Leiden, Netherlands) and 2 μM Pluronic acid (Gibco, Waltham, MA) for 30 min at 37 °C in a medium containing: 135 mM NaCl, 5 mM KCl, 1 mM MgCl_2_, 10 mM HEPES, 10 mM Glucose with an 7,4-adjusted pH (Buffer A) supplemented with 5 mM CaCl_2_. Cells were washed and left to attach in the same buffer on 12 mm Cell-Tak (Corning, NY) precoated coverslides for 20 min, allowing the de-esterification of the dye. Fura-2 was excited alternatively at 340 and 380 nm (Polychrome V, TILL photonics), and fluorescence emission was recorded at 510 nm using a fluorescence microscope (IX71, Olympus) equipped with a dichroic mirror (415DCLP) and a 14-bit CCD camera (ExiBlue, Qimaging). After the stabilization of basal fluorescence, the extracellular medium was replaced with Buffer A supplemented with 0.5 mM CaCl_2_ for 100 s and again with the original 5 mM CaCl_2_-containing Buffer A after curve stabilization. Excitation/emission ratio (F_340nm_/F_380nm_) was calculated for each time point and each cell with the Metafluor 6.3 Software (Universal Imaging, West Chester, USA). The amplitude of CE was calculated after normalization to the basal ratio (ΔF/F_0_), as the difference between average values of basal ratio measured in 5 mM external Ca^2+^ and the average ratio value in 0.5 mM Ca^2+^.

For anti-IgM and Thapsigargin (TG)-induced calcium entry, B cells were loaded in Buffer A containing 1.8 mM CaCl_2_ and 2 μM Fura-2/AM (Fura-2 QBT Kit, Molecular Devices) for 1 h at 37 °C in Cell-Tak precoated 96-well plates, and fluorescence acquisition (excitation 340 and 380 nm; emission 510 nm) was performed on the Flexstation 3 microplate reader with SoftMax Pro 5.4.5 software (Molecular Devices, San Josa, CA). For anti-IgM induced Ca^2+^ response, the extracellular medium was replaced with Buffer A supplemented with 10 mM CaCl_2_, before reading, and 10 μM of polyclonal goat anti-human IgM (Jackson Immunoresearch) were injected after 150 s. TG-induced ER Ca^2+^ release, extracellular medium was replaced with Buffer A supplemented with 100 μM EGTA just before starting the reading protocol. A stimulation with 2 μM of TG was performed after 100 s of recording, and 1.8 mM CaCl_2_ was added after 700 s in order to quantify SOCE entry. Ca^2+^ entry were quantified after value normalization (ΔF/F_0_) with the exception of basal Ca^2+^ concentrations estimated as the average of initial F_340nm_/F_380nm_ values.

In selected experiments, 1 μM Ibrutinib (SelleckChem, Munich, Germany), 5 μM LY294002 (Sigma Aldrich), 2.5 μM Synta66 (Sigma Aldrich), DMSO, 10 μg/ml anti-STIM1 (clone GOK) or 10 μg/ml IgG2a mAb were added to the loading buffer.

### Transient transfection

The small interfering RNAs (siRNA) targeting Orai1 (GCAACGUGCACAAUCUCAAtt, Sigma), STIM1 (AGGUGGAGGUGCAAUAUUAtt, Dharmacon, Lafayette, CO), TRPC1 (s7311), and the control siRNA (4390843) were purchased from Ambion unless specified and transfected in B-CLL cells from 3 CE+ CLL patients by nucleofection (Lonza, Basel, Switzerland) as previously described [17, 21]. After a 48 h-incubation, the knock-down was controlled by flow cytometry and intracellular Ca^2+^ analysis was performed.

### Western blot

Protein extraction was performed by incubating 10^7^ B cells for 30 min on ice in a lysis buffer containing: 20 mM Tris HCl pH 7.5, 150 mM NaCl, 1 mM EDTA, 1 mM EGTA, 1% Triton X100, 2.5 mM Na^+^ pyrosodium tetraphosphate, 1 mM glycerophosphate, 1 mM Na^+^ orthovanadate, 1 μg/ml leupeptin and protease inhibitor cocktail (Roche). Protein extracts were sonicated and centrifuged for 12 min at 16,000 g. The protein concentration of the cell lysates was determined using the micro-bicinchoninic acid assay, and 50 μg were run on SDS-PAGE (10% polyacrylamide gels) in denaturing conditions, and transferred onto PVDF membrane sheets (Bio-Rad, Hercules, CA). After unbound sites were blocked, blots were probed overnight with 5% fat milk in PBS, 0.1% tween 20, and either mouse monoclonal anti-STIM1 (GOK; 1:1000), rabbit polyclonal anti-Orai1 (1:1000; Sigma Aldrich), mouse monoclonal anti-TRPC1 (E6; 1:1000; Santa Cruz, Dallas, TX) or mouse monoclonal anti-GAPDH antibody (6C5; 1:10,000; Abcam, Cambridge, UK). Washed blots were incubated with Horseradish Peroxidase (HRP)-conjugated goat anti-mouse or rabbit IgG Ab (1:10,000; Abcam). Image acquisition was performed on a Chemi-Smart 5100 system (Vilber-Lourmat) with the Chemi-Capt 5000 software and analysed on Image J. All results were normalized upon GAPDH quantification.

### Statistical analysis

Continuous data are described as mean ± standard error of the mean (SEM). Following normality and equality of variance tests, nominal values were compared to controls using the student’s t test or alternatively by using a nonparametric test (Mann-Whitney rank sum test). Differences among groups were analyzed by one-way ANOVA in a non-parametric test and the Dunn’s test was used for post-hoc comparisons. For categorical data the Fisher’s exact test was used, and for correlation analysis the Pearson’s coefficient r was calculated. The profile likelihood method using a Cox regression model of PFS was used in univariate analysis to determine the optimal threshold and stratify patients into two groups as previously described [22]. PFS, TFS and LDT analyses were next performed using Kaplan–Meier curves and prognosis differences between groups were assessed with a log-rank test. Receiver operating curves (ROC) were generated to determine the area under the curve (AUC) and the optimal cut-off values were chosen by using the upper left corner value (100% specificity). *P* values under 0.05 were considered significant. Statistical analyses and the correlation matrix were performed using GraphPad Prism 7.0a (La Jolla, CA).

## Results

### Constitutive Ca^2+^ entry is higher in unstimulated B-CLL cells from patients with progressive disease

**Fig. 1:**
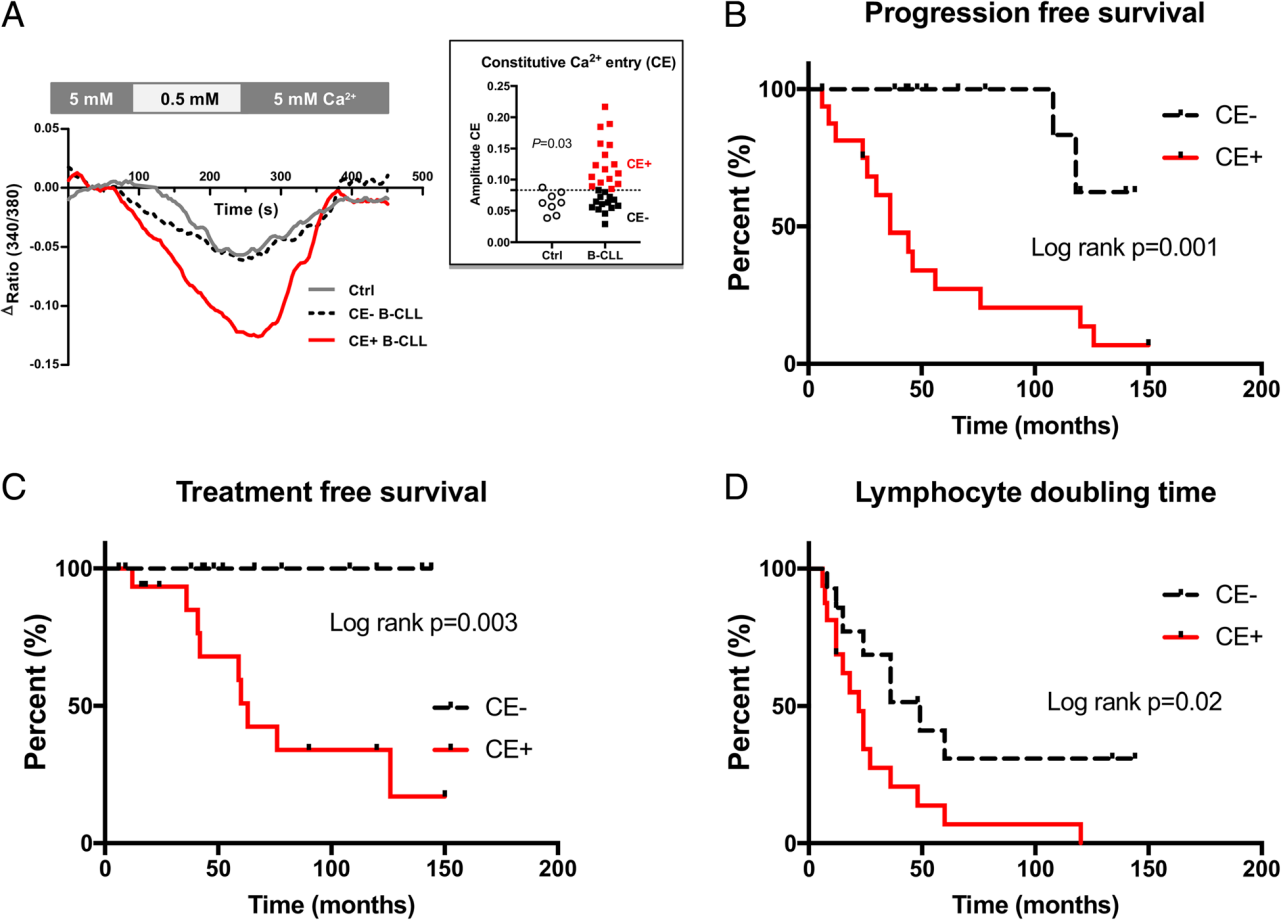
An elevated level of constitutive calcium entry (CE) is relevant for chronic lymphocytic leukemia (CLL) clinical outcome. **a**- Representative kinetic plots of CE in representative healthy control B cells (*n* = 8, Ctrl), CE- B-CLL (*n* = 13) and CE+ B-CLL (*n* = 17) samples. A Cox regression model of progression free survival was used to dichotomize CLL patients in CE- and CE+ (dash line in the islet). Kaplan-Meier plots showing: **b**- time to progression free survival; **c**- time to treatment free survival; and **d**- lymphocyte doubling time between CE+ and CE- CLL groups. *P* values are indicated when significant

As deregulation in Ca^2+^ signaling is an important hallmark of B-CLL cells, and suspected to vary during CLL disease progression [2], Ca^2+^ entry in the absence of BCR engagement, designated as CE, was evaluated in resting B-CLL cells. To this end 30 untreated CLL patients were selected and, as reported in Fig. 1a, CE was significantly enhanced in a subset of B-CLL cells when compared to B cells from 8 healthy controls (ΔF/F_0_: 0.10 ± 0.01 in B-CLL cells versus 0.06 ± 0.01 in controls, *P* = 0.03). CLL patients were further dichotomized into CE+ (high levels) versus CE- (normal/low levels) using the profile likelihood method in a Cox regression model of PFS for optimal cut-off identification (cut-off = 0.083).

Next, and according to this dichotomy, the Kaplan-Meier log-rank analysis revealed, for those CE+ CLL patients (*n* = 16), a significant difference with regards to parameters associated with disease outcome such as PFS (*P* = 0.001; Fig. 1b), TFS (*P* = 0.003; Fig. 1c) and LDT (*P* = 0.02; Fig. 1d). In addition, the Binet status (*p* = 0.0002) and lymphocytosis (*P* = 0.003) were associated with an elevated CE, which was not the case for the cytogenetic risk status, *IgHV* mutational status, and CD38 positivity (Table 1, left part).

**Table 1:** Clinical data of the 30 untreated CLL patients tested for Ca2+ entry and dichotomized into CE+ (high constitutive Ca^2+^ entry [CE]) and CE- (low/normal CE levels) and of the whole CLL cohort (*n* = 74), dichotomized according to the level of STIM1 located in the plasma membrane (STIM1_PM_) in high and low

	CE- (*n* = 14)	CE+ (*n* = 16)	Statistics	High STIM1_PM_ (*n* = 28)	Low STIM1_PM_ (*n* = 44)	Statistics
Age diagnosis (years), mean ± SEM	63 ± 3	62 ± 2	NS	63 ± 2	64 ± 2	NS
Age analysis (years)	69 ± 4	67 ± 2	NS	70 ± 2	70 ± 2	NS
Sex F:M	4:10	8:8	NS	14:14	16:32	NS
Binet A/B/C	12/2/0	2/12/2	0.0002	13/10/5	31/10/3	NS
Cytogenetic risks, No (%)			NS			NS
Low (isolated d13q)	6/12 (50%)	8/16 (50%)		13/26 (50%)	21/37 (56.8%)	
Intermediate (tri12, normal karyotype)	6/12 (50%)	5/16 (31%)		9/26 (34.6%)	13/37 (35.1%)	
High (d11q, d17p, complex karyotype)	0/11 (0%)	3/16 (19%)		4/26 (15.4%)	3/37 (8.1%)	
*IgHV* UM:M	0:7	0:10	NS	3:14	3:21	NS
CD38 (> 30%)	1/12 (8%)	4/15 (27%)	NS	7/28 (25%)	6/45 (13.3%)	NS
Lymphocytosis (Giga/L)	30 ± 4	74 ± 12	0.003	57.2 ± 9.2	34.6 ± 3.5	0.05
PFS (median-months)^a^	> 150	44	0.001	46	120	0.0007
TFS (median-months)^a^	> 150	63	0.003	116	> 120	0.02
LDT (median months)^a^	49	22	0.02	24	36	NS

Abbreviations: *NS* not significant, *No* number, *SEM* standard error of the mean, *IGHV* immunoglobulin heavy-chain variable region, *UM* unmutated *IGHV*, *M* mutated *IGHV*, *PFS* Progression free survival, *TFS* Treatment free survival, *d* deletion, *tri* trisomy, *LDT* Lymphocyte doubling time; ^a^Kaplan-Meyer survival analysis

### Constitutive Ca^2+^ entry is independent from proximal BCR signaling and BCR co-activators

**Fig. 2:**
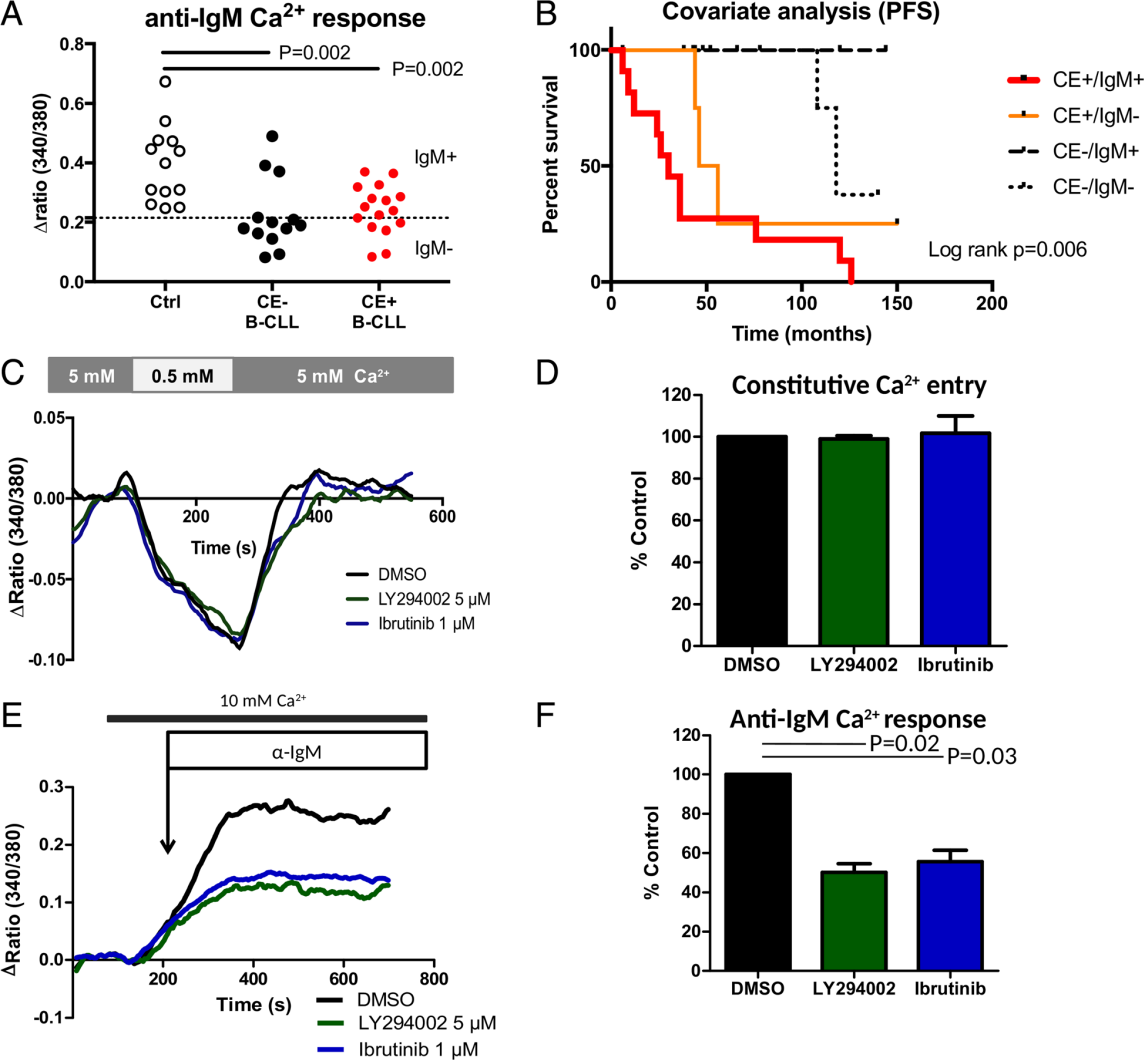
Constitutive calcium entry (CE) is independent from anti-IgM capacity to induce Ca^2+^ signaling in chronic lymphocytic leukemia (CLL). **a**- Normalized ratio of peak to baseline Ca^2+^ flux in response to anti-IgM stimulation of healthy control (*n* = 13, Ctrl), CE- CLL (*n* = 13) and CE+ CLL (*n* = 16) samples. **b**- Bivariate analysis by Kaplan-Meier curves of Ca^2+^ signaling in the context of CLL subsets according to the CE (+/−) and anti-IgM capacity to induce Ca^2+^ signaling (IgM +/−). A Cox regression model of progression free survival (PFS) was used to dichotomize CLL patients in CE- and CE+, on one hand, and IgM- and IgM+, on the other hand. **c/d**- the effects of Ibrutinib (BTK inhibitor, 2.5 μM) and LY294002 (PI3K inhibitor, 5 μM) on CE in CE+/IgM+ B-CLL samples (*n* = 3). **e/f**- the effects of Ibrutinib (BTK inhibitor) and LY294002 (PI3K inhibitor) on anti-IgM capacity to induce Ca^2+^ signaling in CE+/IgM+ B-CLL samples (*n* = 3). *P* values are indicated when significant

One step further, to test BCR pathway dependence in CE+ B-CLL cells, the BCR capacity to mobilize Ca^2+^ was tested within B-CLL cells from 16 CE+ CLL patients, 13 CE- CLL patients, and 13 healthy controls (Fig. 2a and Additional file 2: Figure S2). As previously described [2, 3], Ca^2+^ mobilization in response to BCR engagement was reduced in B-CLL cells when compared to controls (*P* = 0.002 for both CE subgroups), however no difference was observed when comparing the two CE subgroups within CLL patients. Interestingly, by conducting a bivariate analysis of PFS on both CE and IgM Ca^2+^ mobilization, we further observed that CLL patients with disease progression were restricted to CE+/IgM+ (*n* = 11) and CE+/IgM- (*n* = 5) CLL patients but not to CE−/IgM+ (*n* = 4) and CE−/IgM- (*n* = 9) CLL patients (*P* = 0.006, Fig. 2b). To dissect heterogeneity between the 4 subgroups of patients (Additional file 2: Table S1), we next examined whether these differences resulted from differential expression of the membrane surface (s) IgM, sIgD, and co-receptors (CD19, CD21, CD38, and CD5). No differences were observed between the 4 subgroups for these markers that participate or modulate the proximal BCR signaling. As well, no differences were reported when considering CE+ and CE- CLL patients. Accordingly, we concluded that there is independence of CE from proximal BCR signaling and BCR co-activators.

### Constitutive Ca^2+^ entry is independent from an autonomous BCR pathway

Since CE could be attributable to an antigen-independent autonomous BCR pathway [15], this Ca^2+^ entry was recorded in the presence of two BCR signalosome inhibitors Ibrutinib, a covalent inhibitor of BTK, and LY294002, a selective inhibitor of PI3Kδ. As shown in Fig. 2c/d, CE+ B-CLL cells from 3 patients were selected and CE was unaffected by the addition of the BCR signaling inhibitors. In parallel and as a positive control, the Ibrutinib and LY294002 capacity to inhibit Ca^2+^ response following BCR activation was demonstrated (Fig. 2e/f). Such a concept was further reinforced by the analysis of basal pPLCγ2, an indicator of BCR signalosome activation, in resting B-CLL cells showing that pPLCγ2 levels were similar between the CE+ and CE- CLL subgroups (Additional file 2: Table S1).

### Constitutive Ca^2+^ entry is correlated with basal Ca^2+^ levels and independent from SOCE

Next, 29 B-CLL cells (10 CE−/IgM-, 4 CE+/IgM+, 4 CE+/IgM- and 11 CE+/IgM+) were selected and a correlation matrix was performed for all in order to compare CE with (i) the basal intracellular Ca^2+^ level estimated by the initial F340/380 ratio, (ii) the anti-IgM Ca^2+^ response; (iii) the ER Ca^2+^ release by thapsigargin (TG), an inhibitor of the ER Ca^2+^ ATPase pumps, that artificially and maximally deplete Ca^2+^ stores in the absence of extracellular Ca^2+^; and (iv) the TG SOCE response observed after Ca^2+^ reffiling.

Results from the correlation matrix were effective to highlight two groups of Ca^2+^ responses in B-CLL cells (Additional file 2: Figure S3A/B). First an association based on the correlation observed between CE and the basal Ca^2+^ level (r = 0.591; *P* = 0.001), but not CE with anti-IgM and Tg SOCE response (data not shown). Second, a proximal BCR-InsP3R signaling pathway as the anti-IgM Ca^2+^ response was correlated with both TG ER Ca^2+^ release and TG SOCE (*P* = 3 × 10^− 4^ and 1 × 10^− 7^, respectively) but not with basal Ca^2+^ levels and CE.

### Constitutive Ca^2+^ entry is regulated by STIM1and supported by Orai1 and TRPC1 channels

Based on our previous work showing a role for Orai1, TRPC1 channels and STIM1 [17, 23] in CE, and to better characterize the autonomous Ca^2+^ channel influx in CE+ B-CLL cells, three strategies were developed using (1) the Orai1 channel blocker, Synta66 (S66); (2) specific siRNA for Orai1, TRPC1 and STIM1 to modulate CE amplitude; and (3) a quantitative analysis of Orai1, TRPC1 and STIM1 expression by Western-blot.

**Fig. 3:**
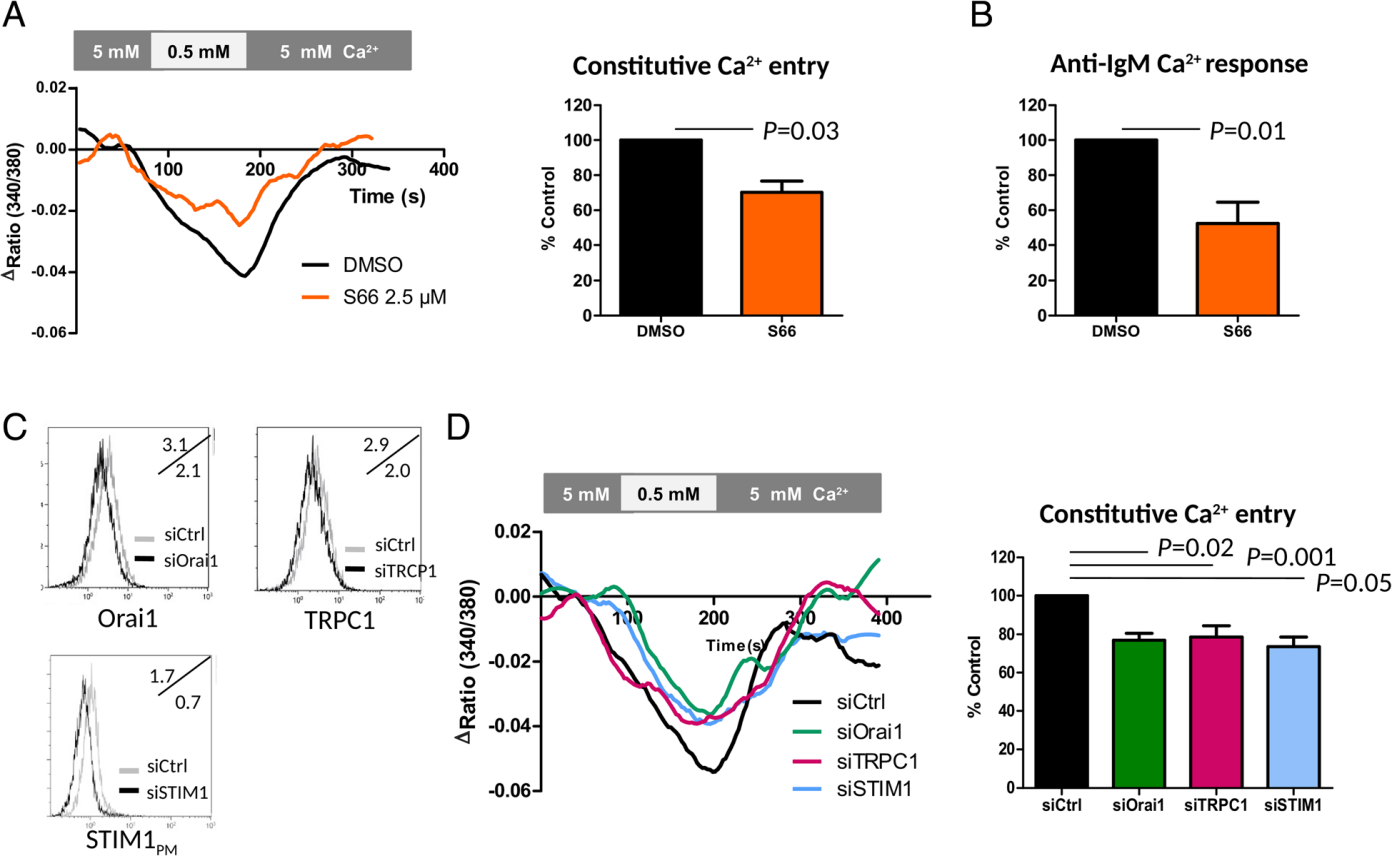
Constitutive calcium entry (CE) is related to STIM1, Orai1 and TRPC1. **a/b**- The effects of the Orai1 channel inhibitor Syntha(S)66 on CE (**a**) and on anti-IgM Ca^2+^ response (**b**) in CE+/IgM+ CLL samples (n = 3). **c**- siRNA control analysis by FACS. The mean fluorescence intensities (MFI) of representative CE+ transfected B-CLL cells are shown in the upper left corner of each plot for the siRNA control, and in the lower left corner for the specific siRNAs. **d**- Average time course of CE in siRNA transfected CE+ B-CLL cells (n = 3). *P* values are indicated when significant

First, specific blockade of Orai1 channels with S66 at 2.5 μM significantly reduced CE (*P* = 0.03), the anti-IgM Ca^2+^ response (*P* = 0.01), and TG SOCE (*P* = 0.05) but not TG ER Ca^2+^ release in CE+/IgM+ B-CLL cells compared to control conditions (Fig. 3a/b and data not shown). Second, and another way in which to further test our hypothesis, was to reduce the expression of Orai1, TRPC1 and/or STIM1 by transfecting specific siRNA into B-CLL cells (1 CE+/IgM- and 2 CE+/IgM+). In contrast to the negative siRNA control, a reduction was seen at the protein level when using specific siRNAs for STIM1, Orai1, and TRPC1 (FACS representations are depicted Fig. 3c). As a result, CE was reduced in the presence of siRNA to Orai1, TRPC1 and STIM1 (*P* < 0.05 for all) (Fig. 3d). These results suggest that Orai1 together with TRPC1 both contribute to CE regulated by STIM1.

**Fig. 4:**
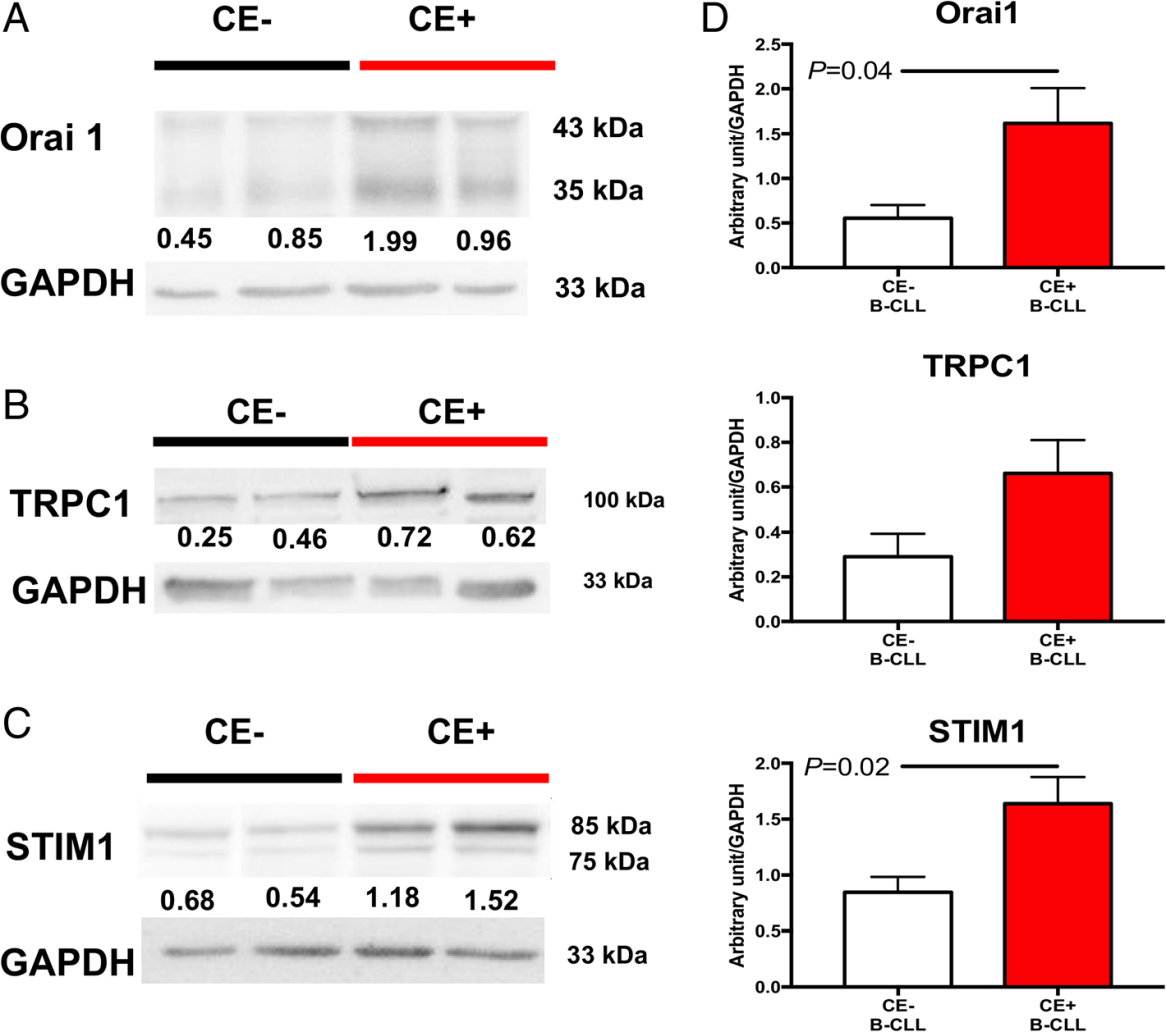
B-CLL cells from CE+ CLL patients (*n* = 11) display increased Orai1 and STIM1 protein levels compared with those from CE- CLL patients (*n* = 8). Left panels, representative Western blot images for Orai1(**a**), TRPC1 (**b**), and STIM1 (**c**). Quantification after normalization to GAPDH protein expression is depicted (**d**). *P* values are indicated when significant

Third, Western blot (WB) was used to analyze the expression of Orai1, STIM1 and TRPC1 isoforms in B-CLL cells from 19 patients (11 CE+ and 8 CE-). When comparing CE+ and CE- patients (Fig. 4), the two different isoforms of Orai1 were increased (*P* = 0.04), and, although not significant, there is a trend for higher TRPC1 expression in CE+ B-CLL cells compared to CE- B-CLL cells. STIM1 analysis by WB reveals higher expression of both the 75 kDa non-glycosylated isoform and the 85 kDa glycosylated isoform that were overexpressed in CE+ B-CLL cells (*P* = 0.03).

### The pool of STIM1 located in the plasma membrane (STIM1_PM_) controls CE

**Fig. 5:**
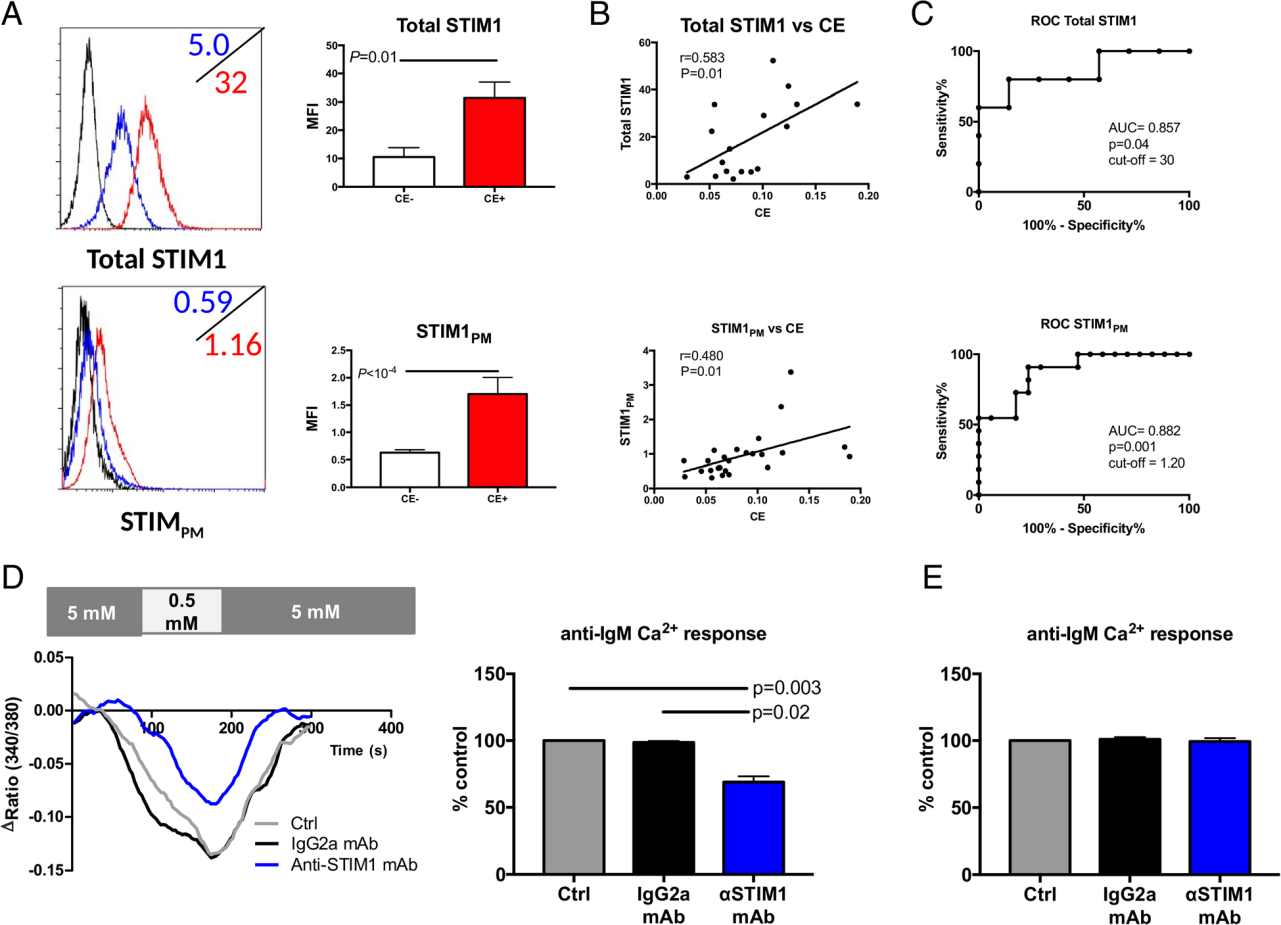
The pool of STIM1 in plasma membrane controls constitutive calcium entry (CE). **a**- Cytoplasmic (after permeabilization, total STIM1[t]) and plasma membrane staining of STIM1 (STIM1_PM_) on B-CLL cells from CE+ (*n* = 7) and CE- (*n* = 12) CLL patients. The isotype control is shown (black line). The mean fluorescence intensities (MFI) of representative CE- B-CLL cells are shown in the upper left corner of each plot in blue, and in the lower left corner in blue for CE+ B-CLL cells. **b**- Correlation between CE and tSTIM1_T_ (upper panel) or STIM1_PM_ (lower panel). **c**- Receiver operating curves (ROC) were generated to determine the area under the curve (AUC) and the optimal cut-off value to discriminate STIM1 high from STIM1 low patients. **d**- The effects of the anti-STIM1 mAb clone GOK on CE in CLL samples (*n* = 6). **e**- No effect of the anti-STIM1 mAb on anti-IgM Ca^2+^ response in CLL samples (*n* = 10). The r^2^ coefficient and *P* values are indicated when significant

Since glycosylation is required for STIM1 localization at the plasma membrane [24, 25], and given that the pool of STIM1 located in the plasma membrane (STIM1_PM_) regulates store-independent Ca^2+^ influx [26], this raises the possibility that STIM1_PM_ controls CE and contributes to its enhancement in CE+ B-CLL cells. To address this issue (Fig. 5a), B-CLL cells from 28 patients (11 CE+ and 17 CE-) were tested by FACS for STIM1 expression using STIM1 mAb following permeabilization of the cells (total-STIM1 expression determination) or not (STIM1_PM_ quantification). In agreement with WB results, FACS analysis revealed that both total-STIM1 and STIM1_PM_ were increased in CE+ B-CLL cells (*P* = 0.01 and < 10^− 4^, respectively), and their levels correlated with CE amplitude (*P* = 0.01 both, Fig. 5b). A ROC analysis was performed in order to establish the cut-off for positivity (Fig. 5b left). We next sought to determine STIM1_PM_ involvement in CE regulation, and this was tested by exploring the capacity of the anti-STIM1 mAb (GOK, 10 μg/mL) to inhibit CE. In contrast to the IgG2a isotype control mAb that had no effect on CE (Fig. 5c), the anti-STIM1 mAb inhibits CE (*P* = 0.03), while no effects were reported on the anti-IgM Ca^2+^ response, TG ER Ca^2+^ release and TG SOCE responses (Fig. 5d and Additional file 2: Figure S4B). This is in agreement with the observed correlation between STIM1_PM_ levels and basal Ca^2+^ but not with TG ER Ca^2+^ release and IgM/TG SOCE results (Additional file 2: Figure S4A). Altogether this reinforces our hypothesis that CE and basal Ca^2+^ are regulated by STIM1_PM_ and supported by Orai1 and TRPC1 channels in a unique and alternative influx pathway distinct from SOCE and downstream the BCR-InsP3R pathway.

### STIM1_PM_ as a valuable therapeutic target

**Fig. 6:**
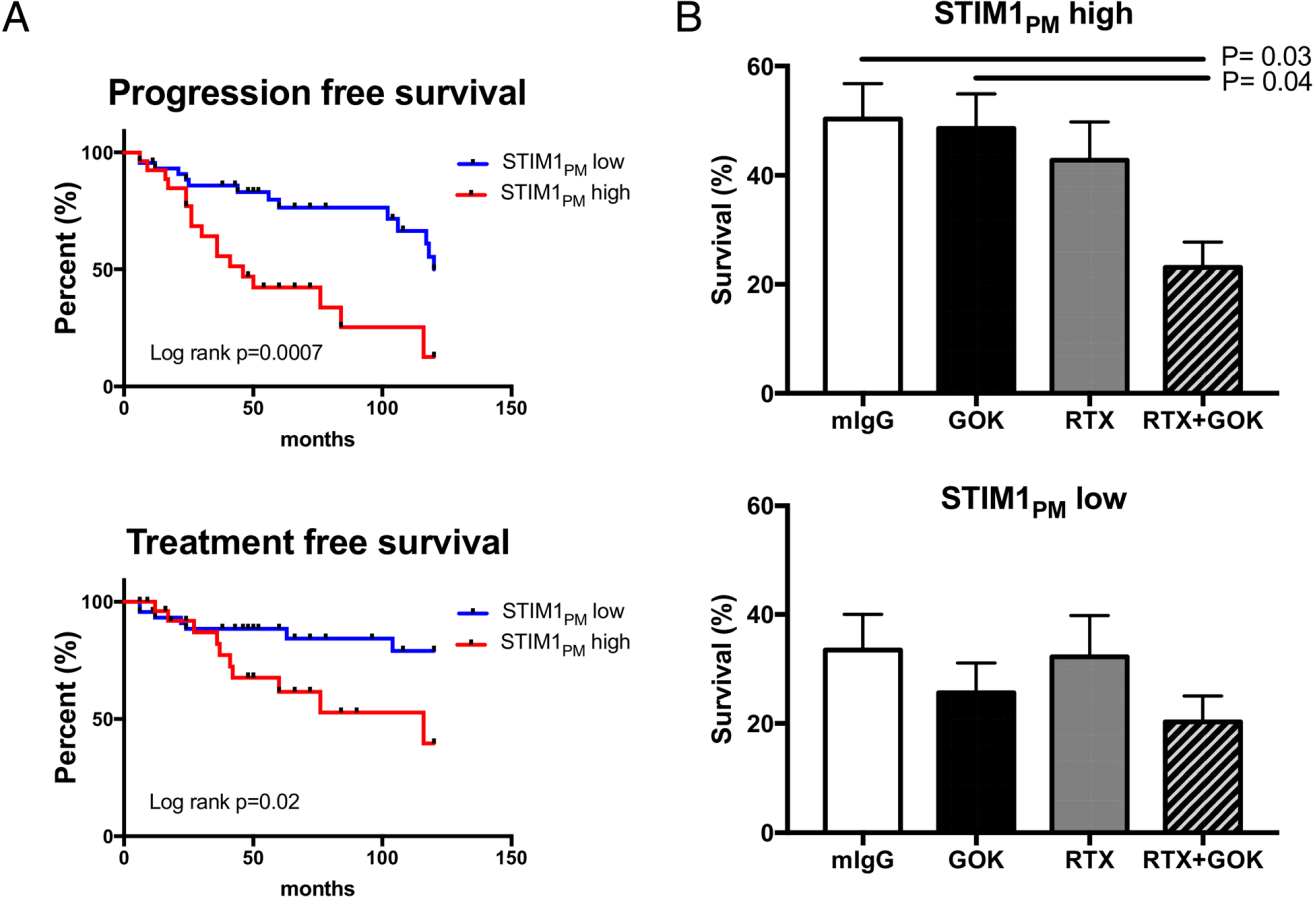
In the whole CLL cohort (*n* = 74), an elevated level of STIM1 at plasma membrane (STIM1PM) is relevant for CLL clinical outcome and influence in vitro cell survival. **a** Kaplan-Meier plots showing progression free survival and treatment free survival for STIM1PM dichotomize into high and low levels. **b** Increase in the density of STIM1PM improves the efficacy of rituximab (RTX) in the STIM1_PM_ high CLL subgroup (*n* = 9) when used in combination with the anti-STIM1 mAb (both 10 μg/mL, 48 h), effect which was not observed in the STIM1_PM_ low CLL subgroup (*n* = 8). P values are indicated when significant

As CE determination is difficult to manage in routine practice, we further compared the patient’s characteristics according to their plasma membrane STIM1 status in 74 untreated CLL that included those tested for Ca^2+^ signaling. As depicted in the Kaplan-Meyer curves (Fig. 6a), the CLL STIM1_PM_ high subgroup had shorter PFS and TFS (*P* = 0.0007 and *P* = 0.02, respectively). Characteristics of STIM1_PM_ high and low patients are presented in Table 1 (right part) showing that lymphocytosis (*p* = 0.05), but not the other parameters tested, was increased in the CLL STIM1_PM_ high subgroup.

Finally and as the initial descriptions of STIM1_PM_ were related to the control of cell survival [27, 28], we next decided to test the neutralizing capacity of the anti-STIM1 mAb clone GOK to control B-CLL cell survival (STIM1_PM_ high *n* = 9; and STIM1_PM_ low *n* = 8) when used alone or in combination with RTX, an anti-CD20 mAb (Fig. 6b). Used alone GOK and RTX did not reduce in vitro B-CLL cell survival as compared to the controls, but in contrast the RTX + GOK combination significantly reduced cell viability in the STIM1_PM_ high subgroup (50.4 ± 6.4% with IgG2a versus 23.0 ± 4.7% with RTX + GOK, *P* = 0.03), an effect which was not significant in the STIM1_PM_ low subgroup (33.5 ± 6.5% with IgG2 versus 20.3 ± 4.7% with RTX + GOK).

## Discussion

The overall data add new support to the critical role played by the Ca^2+^ signaling in CLL outcome, and describe for the first time a novel STIM1_PM_-dependant and constitutively active Ca^2+^ entry, independent from BCR signaling, and that constitutively active CE can be modulated and targeted by an anti-STIM1 mAb. We found that both CE and STIM1_PM_ are clinically relevant in CLL and their determinations present important prognostic value.

Several reports have demonstrated altered Ca^2+^ signaling in CLL B cells and with the paradox that Ca^2+^ mobilization is altered in “anergic” CLL B cells from non-progressive patients, while a response is reported in CLL B cells from patients with disease progression as observed in our study [2, 3]. Moreover and based on the strong correlation observed between CE and basal cytosolic Ca^2+^ concentrations in this study, we were able to extend the observation performed by Muggen and colleagues who have described elevated basal Ca^2+^ concentrations in B-CLL cells in contrast to normal B cells [14]. Our study also supports that CE and the elevated level of basal Ca^2+^ reported in B-CLL cells are, in fact, independent from the BCR-PLCγ2-InsP_3_R pathway and are instead related to an enhanced CE and are independent from store depletion. In contrast, Duhren-Von Minden and colleagues have associated the elevated basal Ca^2+^ signaling downstream Syk phosphorylation in CLL B cells to an antigen-independent recognition of the BCR framework domains (FR2 or FR3), or alternatively through an occupation of the BCR with repetitive motifs [15]. Importantly, blocking the BCR pathway with the BTK inhibitor ibrutinib or with the PI3K inhibitor LY294002 did not alter CE or the basal Ca^2+^ level (data not shown) which is in agreement with Muggen report who failed to associate the basal Ca^2+^ level in CLL B cells with the FR2/3 amino-acid sequence. Based on the report of Le Roy and colleagues who detected pSyk at a basal level in IgM+ responder patients, it could be proposed that blocking pSYK controls both CE and the IgM response in CE+/IgM+ responder patients, an hypothesis that needs to be tested as well as the capacity of Syk to phosphorylate STIM1 [2, 3].

STIM1 was initially identified as a plasma membrane protein [25], and more recently STIM1_PM_ was associated with the regulation of a store independent Ca^2+^ entry pathway activated by arachidonic acid [26] and to SOCE in platelets [29]. Similarly and although STIM1 is predominantly located in the ER in normal B cells, we found that CE+ B-CLL cells express a substantial amount of STIM1_PM_ and Orai1 as well as an enhanced expression of TRPC1. This is important because STIM1_PM_ can interact with Orai1 or TRPC1, two Ca^2+^ channels activated in CE+ B-CLL cells as demonstrated by using specific siRNAs and in agreement with the Chen KT et al. report [30]. STIM1 deregulation in B-CLL cells needs further exploration as it may be related to defective transcriptional control by DNA methylation and/or microRNAs [31, 32], and/or is related to post-translational modifications such as glycosylation and/or phosphorylation known to affect STIM1 localization and properties [24, 33], as these processes are altered during CLL evolution [34].

The clinical success of RTX in monotherapy is limited in CLL and, in order to improve its efficacy, RTX is associated with chemotherapy (RFC) or with BCR inhibitors (Ibrutinib, Idelalisib, venetoclax), however relapses and side-effects remain important suggesting a need to develop new therapeutical options and in particular to combine RTX with new drugs targeting a non BCR survival pathway [35, 36]. Consistent with the notion that CE is important for disease outcome and STIM1_PM_ for CE, we demonstrated that pre-incubating cells with antibodies targeting STIM1_PM_ reverses B-CLL cell capacity for CE and in turn impairs cell survival when associated with RTX. Therefore, we propose to use anti-STIM1 mAb targeting STIM1_PM_ and CE as a new innovative therapeutic option for CLL. An additive/synergic effect of RTX or BCR inhibitors with CE inhibitors, such as anti-STIM1 mAb, should be addressed in future studies.

Relevant limitations of our study include the following: (i) a small sample size used to analyze Ca^2+^ entry in CLL B cells; (ii) the use of samples from a cross-sectional and monocentric center; and (iii) a bias due to the selection of untreated patients. However and to reduce these limitations, a large panel of approaches (e.g. Ca^2+^ signaling, siRNAs, specific inhibitors, FACS, WB) has been used in order to demonstrate that STIM1 and in particular STIM1_PM_ controls CE in CLL B cells from patients with progressive disease. The selection of untreated patients for this study represents also an advantage as drug exposure may affect the analysis of Ca^2+^ entry, as observed in vitro with ibrutinib. Future studies are however mandatory in order to study whether variations in Ca^2+^ entry and Ca^2+^ actor variations vary following treatment introduction and in those patients who relapse.

## Conclusion

In CLL the involvement of Ca^2+^ signaling deregulation in cancer cell progression is well established, but the identification of mechanisms controlling Ca^2+^ entry are poorly understood. In the present work, an extensive analysis of the Ca^2+^ entry in CLL cells was performed, revealing, in patients with progressive disease, the implication of a constitutive and BCR-independent Ca^2+^ entry pathway. Next, it was further observed that a pool of STIM1 present in the plasma membrane characterizes tumor progression and controls constitutive Ca^2+^ entry. Finally, the capacity of an anti-STIM1 mAb to block constitutive Ca^2+^ entry and to reduce in vitro CLL cell viability, when associated with Rituximab, was reported within the high STIM1_PM_ CLL subgroup. This supports the idea that targeting STIM1_PM_ and therefore constitutive Ca^2+^ entry represents a new 1st in class therapeutic pathway in leukemia treatment. The potential use of mAb targeting STIM1_PM_ in cancer therapy that can be used alone or in synergy with existing drugs needs to be further evaluated.

## Additional files

Additional file 1: **Figure S1.** Two pathways control Ca^2+^ signaling in B cells from patients with chronic lymphocytic leukemia. In the BCR-induced store operated Ca^2+^ entry pathway, B cell receptor (BCR) interaction with the antigen results in the formation of the signalosome consisting of an active complex composed of the tyrosine kinases Lyn and Syk, B-cell linker protein (BLNK), Bruton-tyrosine-kinase (BTK), phospholipase C gamma 2 (PLCγ2), and phosphatidylinositol-4,5-bisphosphate 3-kinase δ (PI3Kδ) that phosphorylates CD19. Signalosome activation cleaves the membrane phospholipid phosphatidyl inositol 4.5-biphosphate (InsP2) into diacylglycerol (DAG) and inositol 1,4,5-triphosphate (InsP3), which subsequently, through binding to the endoplasmic reticulum (ER) IP3 receptor (InsP_3_R), mobilizes initially Ca^2+^ from stores and secondarily extracellular Ca^2+^ through the interaction between the multimerized reticular stromal interaction molecule 1 (STIM1_ER_) and the plasma-membrane Orai1 channel. In the constitutive Ca^2+^ pathway, Ca^2+^ entry is triggered by STIM1 located at the plasma-membrane (STIM1_PM_). (PPTX 90 kb)

Additional file 2 **Table S1.** B cell receptors (BCR) and co-receptors analysis in B-CLL cells (*n* = 30) according to the capacity of the cells to possess an elevated constitutive Ca2+ entry (CE+) and their capacity to mobilize Ca2+ in response to BCR engagement (IgM+). The phosphorylated phospholipase Cγ2 (pPLCγ2), an immediate downstream BCR effector, was added to the list. **Figure S2.** Representative kinetic plots of anti-IgM Ca^2+^ response (A), and thapsigargin (TG) Ca^2+^ response (B) in representative healthy donor (HD) control B cells (*n* = 8), CE- B-CLL (*n* = 13) and CE+ B-CLL (*n* = 16) samples. Cox regression model of progression free survival (PFS) was used to dichotomize CLL patients in IgM- and IgM+ (dash line). From the TG Ca^2+^ response analysis, the basal Ca^2+^ level was evaluated before normalization (1), the TG capacity to release Ca^2+^ from the endoplasmic reticulum (ER), and the TG capacity to release SOCE following extracellular medium supplementation with 1.8 mM Ca^2+^. *P* values are indicated when significant. **Figure S3.** Basal calcium (Ca2+) entry is related to constitutive calcium entry (CE) but not to store operated Ca2+ entry (SOCE), while the anti-IgM Ca2+ response correlated to thapsigargin (TG) capacity to induce endoplasmic reticulum (ER) Ca2+ release and SOCE. **Figure S4.** The pool of STIM1 in plasma membrane (STIM1_PM_) is correlated with basal Ca^2+^ levels but independent from anti-IgM Ca^2+^ response and thapsigargin (TG) capacity to release Ca^2+^ from the endoplasmic reticulum (ER) and to induce SOCE. Correlations between STIM1_PM_ levels with basal Ca^2+^ (A), anti-IgM Ca^2+^ response (B), TG capacity to induce ER Ca^2+^ release (C), and TG SOCE (D). Values were obtained from 18 CLL, see material and methods for details. *P* and r^2^ values are indicated when significant. (DOCX 531 kb)

## Abbreviations

BCR: B cell receptor
Ca^2+^: Calcium
CE: Constitutive Ca^2+^ entry
CLL: Chronic lymphocytic leukemia
ER: Endoplasmic reticulum
FACS: Fluorescence-activated cell sorting
InsP3: Inositol 1,4,5-triphosphate
InsP_3_R: Inositol 1,4,5-triphosphate receptor
LDT: Lymphocyte doubling time
mAb: Monoclonal antibody
mAb: Monoclonal antibody
PFS: Progression free survival
PLCγ2: Phospholipase C gamma 2
RTX: Rituximab
siRNA: Small interfering RNA
SOCE: Store operated Ca^2+^ entry
STIM1: Stromal interaction molecule 1
STIM1_PM_: Plasma-membrane STIM1
TFS: Treatment free survival
TG: Thapsigargin
WB: Western blot
