# Supplementary material for: STIM1 at the plasma membrane as a new target in progressive chronic lymphocytic leukemia
Source: J Immunother Cancer. 2019 Apr 23;7:111. doi: 10.1186/s40425-019-0591-3 (PMC6480884; doi:10.1186/s40425-019-0591-3)
Supplement: Supplementary file 1 — Figure S1. Two pathways control Ca2+ signaling in B cells from patients with chronic lymphocytic leukemia. In the BCR-induced store operated Ca2+ entry pathway, B cell receptor (BCR) interaction with the antigen results in the formation of the signalosome consisting of an active complex composed of the tyrosine kinases Lyn and Syk, B-cell linker protein (BLNK), Bruton-tyrosine-kinase (BTK), phospholipase C gamma 2 (PLCγ2), and phosphatidylinositol-4,5-bisphosphate 3-kinase δ (PI3Kδ) that phosphorylates CD19. Signalosome activation cleaves the membrane phospholipid phosphatidyl inositol 4.5-biphosphate (InsP2) into diacylglycerol (DAG) and inositol 1,4,5-triphosphate (InsP3), which subsequently, through binding to the endoplasmic reticulum (ER) IP3 receptor (InsP3R), mobilizes initially Ca2+ from stores and secondarily extracellular Ca2+ through the interaction between the multimerized reticular stromal interaction molecule 1 (STIM1ER) and the plasma-membrane Orai1 channel. In the constitutive Ca2+ pathway, Ca2+ entry is triggered by STIM1 located at the plasma-membrane (STIM1PM). (PPTX 90 kb) [file 40425_2019_591_MOESM1_ESM.pptx]

## Slide 1
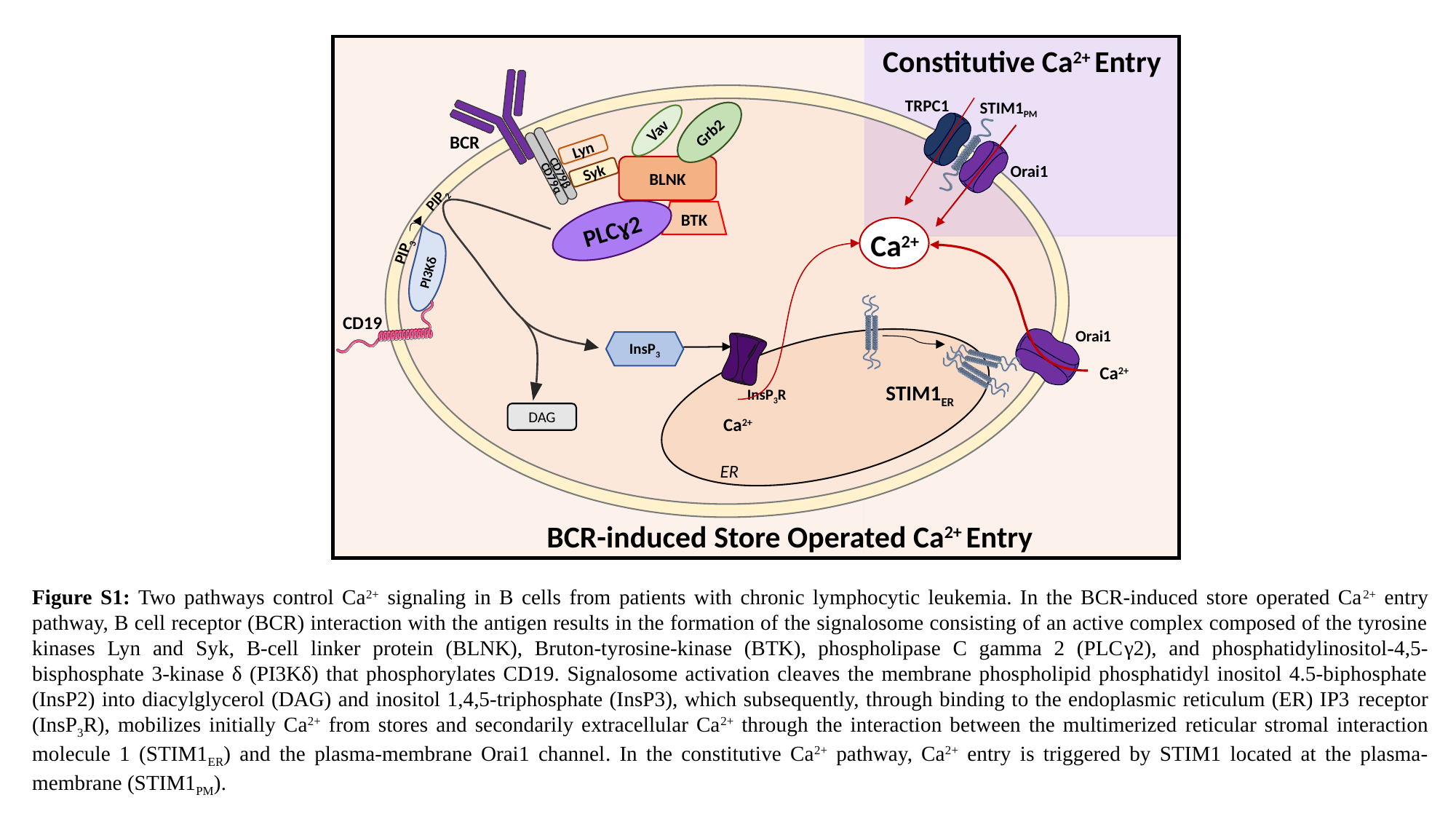

Constitutive Ca2+ Entry
TRPC1
STIM1PM
Grb2
Vav
CD79β
CD79α
BCR
Lyn
Orai1
BLNK
Syk
PIP2
BTK
PLCɣ2
Ca2+
PIP3
PI3Kδ
CD19
Orai1
InsP3
STIM1ER
InsP3R
DAG
ER
BCR-induced Store Operated Ca2+ Entry
Ca2+
Ca2+
Figure S1: Two pathways control Ca2+ signaling in B cells from patients with chronic lymphocytic leukemia. In the BCR-induced store operated Ca2+ entry pathway, B cell receptor (BCR) interaction with the antigen results in the formation of the signalosome consisting of an active complex composed of the tyrosine kinases Lyn and Syk, B-cell linker protein (BLNK), Bruton-tyrosine-kinase (BTK), phospholipase C gamma 2 (PLCγ2), and phosphatidylinositol-4,5-bisphosphate 3-kinase δ (PI3Kδ) that phosphorylates CD19. Signalosome activation cleaves the membrane phospholipid phosphatidyl inositol 4.5-biphosphate (InsP2) into diacylglycerol (DAG) and inositol 1,4,5-triphosphate (InsP3), which subsequently, through binding to the endoplasmic reticulum (ER) IP3 receptor (InsP3R), mobilizes initially Ca2+ from stores and secondarily extracellular Ca2+ through the interaction between the multimerized reticular stromal interaction molecule 1 (STIM1ER) and the plasma-membrane Orai1 channel. In the constitutive Ca2+ pathway, Ca2+ entry is triggered by STIM1 located at the plasma-membrane (STIM1PM).
