# Supplementary material for: STIM1 at the plasma membrane as a new target in progressive chronic lymphocytic leukemia
Source: J Immunother Cancer. 2019 Apr 23;7:111. doi: 10.1186/s40425-019-0591-3 (PMC6480884; doi:10.1186/s40425-019-0591-3)
Supplement: Supplementary file 2 — Table S1. B cell receptors (BCR) and co-receptors analysis in B-CLL cells (n = 30) according to the capacity of the cells to possess an elevated constitutive Ca2+ entry (CE+) and their capacity to mobilize Ca2+ in response to BCR engagement (IgM+). The phosphorylated phospholipase Cγ2 (pPLCγ2), an immediate downstream BCR effector, was added to the list. Figure S2. Representative kinetic plots of anti-IgM Ca2+ response (A), and thapsigargin (TG) Ca2+ response (B) in representative healthy donor (HD) control B cells (n = 8), CE- B-CLL (n = 13) and CE+ B-CLL (n = 16) samples. Cox regression model of progression free survival (PFS) was used to dichotomize CLL patients in IgM- and IgM+ (dash line). From the TG Ca2+ response analysis, the basal Ca2+ level was evaluated before normalization (1), the TG capacity to release Ca2+ from the endoplasmic reticulum (ER), and the TG capacity to release SOCE following extracellular medium supplementation with 1.8 mM Ca2+. P values are indicated when significant. Figure S3. Basal calcium (Ca2+) entry is related to constitutive calcium entry (CE) but not to store operated Ca2+ entry (SOCE), while the anti-IgM Ca2+ response correlated to thapsigargin (TG) capacity to induce endoplasmic reticulum (ER) Ca2+ release and SOCE. Figure S4. The pool of STIM1 in plasma membrane (STIM1PM) is correlated with basal Ca2+ levels but independent from anti-IgM Ca2+ response and thapsigargin (TG) capacity to release Ca2+ from the endoplasmic reticulum (ER) and to induce SOCE. Correlations between STIM1PM levels with basal Ca2+ (A), anti-IgM Ca2+ response (B), TG capacity to induce ER Ca2+ release (C), and TG SOCE (D). Values were obtained from 18 CLL, see material and methods for details. P and r2 values are indicated when significant. (DOCX 531 kb) [file 40425_2019_591_MOESM2_ESM.docx]

STIM1 at the plasma membrane as new target in progressive chronic lymphocytic leukemia

**Short title:** constitutive calcium entry in chronic lymphocytic leukemia

**Table S1:** B cell receptors (BCR) and co-receptors analysis in B-CLL cells (n=30) according to the capacity of the cells to possess an elevated constitutive Ca2+ entry (CE+) and their capacity to mobilize Ca2+ in response to BCR engagement (IgM+). The phosphorylated phospholipase Cγ2 (pPLCγ2), an immediate downstream BCR effector, was added to the list.

|  | **CE-/IgM-** | **CE-/IgM+** | **CE+**  **/IgM-** | **CE+**  **/IgM+** | **ANOVA** | **CE-** | **CE+** | **t test** |
| --- | --- | --- | --- | --- | --- | --- | --- | --- |
| **sIgM** | 4.0±1.6 | 3.4±0.8 | 2.9±1.7 | 2.4±0.6 | NS | 3.7±0.9 | 2.5±1.9 | NS |
| **sIgD** | 3.3±1.1 | 3.7±1.2 | 4.3±1.4 | 3.7±0.8 | NS | 3.5±0.8 | 3.8±0.7 | NS |
| **CD19** | 6.7±1.1 | 5.2±0.9 | 9.2±1.9 | 6.0±0.7 | NS | 6.0±0.7 | 6.9±0.8 | NS |
| **CD21** | 1.2±0.4 | 1.0±0.1 | 1.8±0.4 | 1.2±0.3 | NS | 1.1±0.3 | 1.4±0.2 | NS |
| **CD38** | 4.3±1.0 | 20±16.7 | 19.2±10.3 | 18.5±7.4 | NS | 9.0±5.2 | 17.9±5.5 | NS |
| **CD5** | 5.2±0.7 | 5.0±1.0 | 4.9±0.8 | 4.9±0.8 | NS | 5.2±0.6 | 4.9±0.6 | NS |
| **pPLCγ2** | 0.8±1.1 | 1.0±0.1 | 0.9±0.2 | 1.0±0.1 | NS | 0.9±0.1 | 1.0±0.1 | NS |

Results are expressed in mean fluorescence intensity (MFI) except for CD38 that was expressed in percentage (%) of positive B-CLL cells.

**Figure S2:** Representative kinetic plots of anti-IgM Ca^2+^ response (**A**), and thapsigargin (TG) Ca^2+^ response (**B**) in representative healthy donor (HD) control B cells (n=8), CE- B-CLL (n=13) and CE+ B-CLL (n=16) samples. Cox regression model of progression free survival (PFS) was used to dichotomize CLL patients in IgM- and IgM+ (dash line). From the TG Ca^2+^ response analysis, the basal Ca^2+^ level was evaluated before normalization (1), the TG capacity to release Ca^2+^ from the endoplasmic reticulum (ER), and the TG capacity to release SOCE following extracellular medium supplementation with 1.8 mM Ca^2+^. *P* values are indicated when significant.


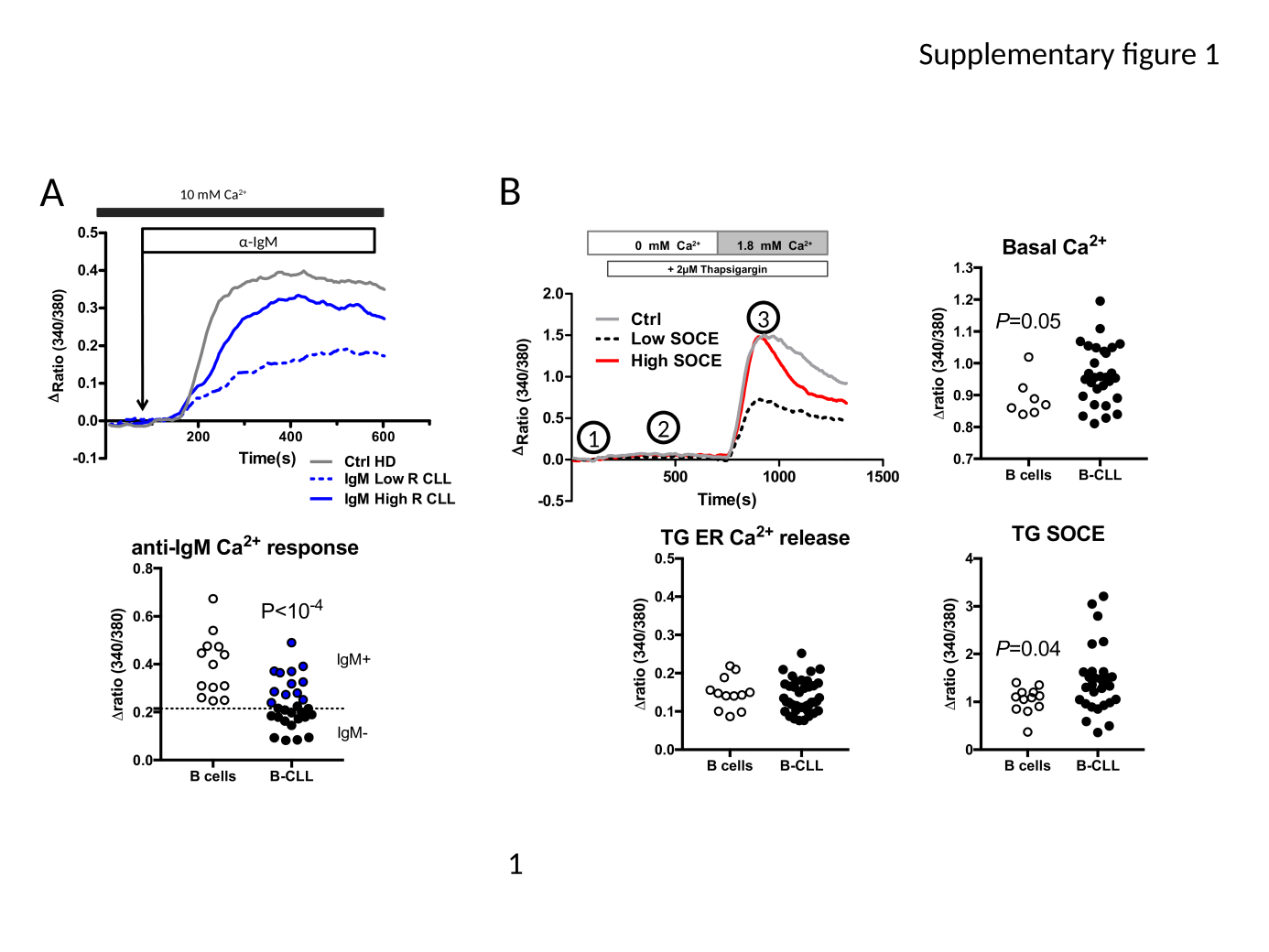


**Figure S3: Basal calcium (Ca2+) entry is related to constitutive calcium entry (CE) but not to store operated Ca2+ entry (SOCE), while the anti-IgM Ca2+ response correlated to thapsigargin (TG) capacity to induce endoplasmic reticulum (ER) Ca2+ release and SOCE. A:** Correlation matrix between Ca2+ entry parameters including CE, basal Ca2+, anti-IgM Ca2+ response (αIgM), TG capacity to induce ER Ca2+ release, and TG SOCE. Values were obtained from 29 CLL patients, see material and methods for details. **B:** CE and basal Ca2+ levels, on one hand, and anti-Ig-M and TG SOCE responses are correlated, on the other hand. P and r2 values are indicated when significant.

**
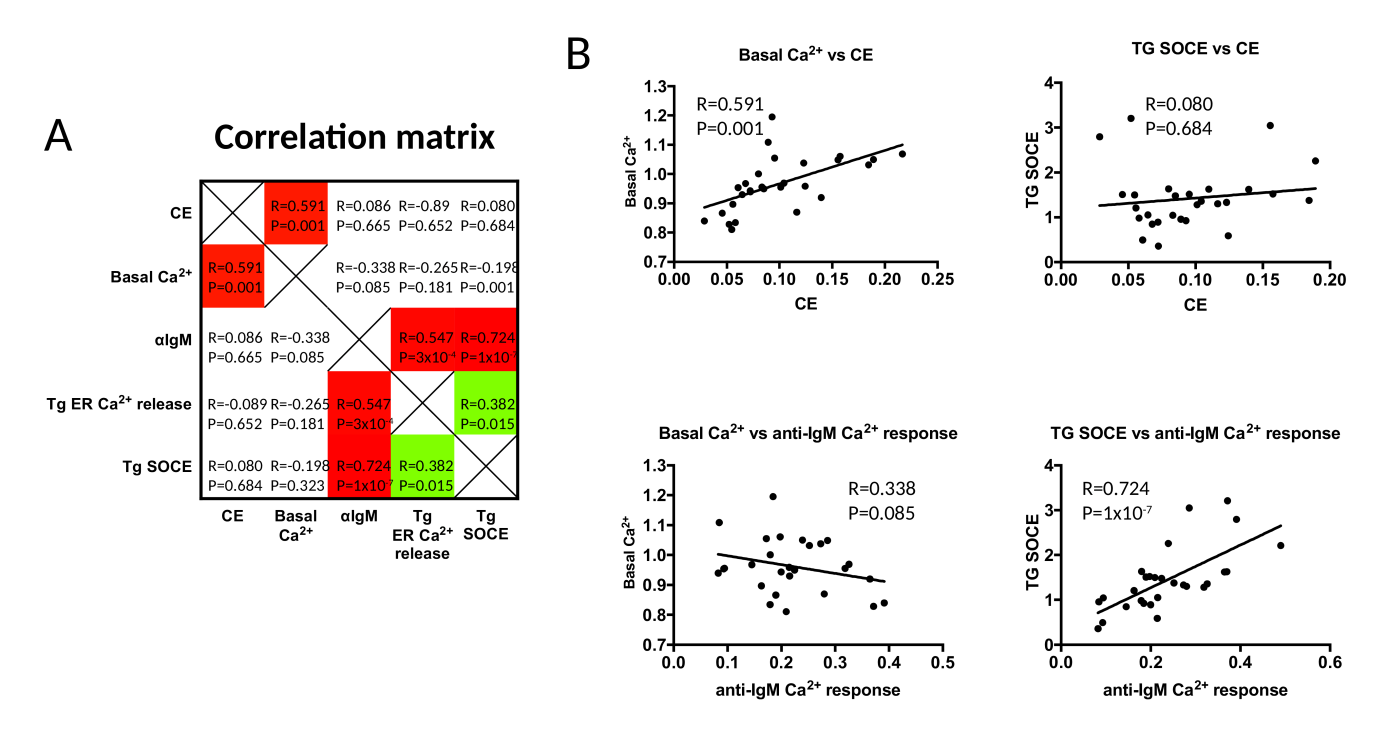
**

**Figure S4:** The pool of STIM1 in plasma membrane (STIM1_PM_) is correlated with basal Ca^2+^ levels but independent from anti-IgM Ca^2+^ response and thapsigargin (TG) capacity to release Ca^2+^ from the endoplasmic reticulum (ER) and to induce SOCE. Correlations between STIM1_PM_ levels with basal Ca^2+^ (A), anti-IgM Ca^2+^ response (B), TG capacity to induce ER Ca^2+^ release (C), and TG SOCE (D). Values were obtained from 18 CLL, see material and methods for details. *P* and r^2^ values are indicated when significant.
